# Hepatocellular carcinoma presenting as spinal cord compression in Native Americans with controlled hepatitis C: two case reports

**DOI:** 10.1186/s13256-018-1807-8

**Published:** 2018-09-30

**Authors:** Maksim Liaukovich, Susan Wu, Sydney Yoon, Jeff Schaffer, Jen C. Wang

**Affiliations:** 1 Division of Hematology/Oncology, Brookdale University Hospital Medical Center, Brooklyn, NY 11212 USA; 2 Department of Pathology, South Naussau Communities Hospital, Oceanside, NY USA; 3 Department of Radiology, South Naussau Communities Hospital, Oceanside, NY USA; 4 Department of Cardiology, South Naussau Communities Hospital, Oceanside, NY USA

**Keywords:** Hepatocellular carcinoma, Hepatitis C, Native American

## Abstract

**Background:**

Hepatocellular carcinoma is a common malignancy in Asia. It is associated with chronic hepatitis B virus or hepatitis C virus infection and alcoholic hepatitis. Commonly, the tumor metastasizes to the lungs, regional lymph nodes, and bone. Recently, the incidence of metastatic spinal cord compression caused by primary hepatocellular carcinoma has been reported more frequently due to improved diagnosis and therapeutic modalities. The presentation of primary hepatocellular carcinoma with spinal cord compression is very rare. To the best of our knowledge, there are only 33 such cases published to date. The majority of cases involve patients of Asian origin and are associated with hepatitis B infection.

**Case presentation:**

We report consecutive cases of two Native American (American Indian) patients (a 64-year-old man and a 70-year-old man) who presented with symptoms of spinal cord compression due to metastatic spread of hepatocellular carcinoma and were associated with hepatitis C infection. In one of these cases, the hepatitis C infection had been successfully controlled (hepatitis C titers were undetectable for 1 year before he presented with spinal cord compression). This occurrence in a Native American with a controlled hepatitis C infection has not been reported previously.

**Conclusions:**

Primary care physicians, oncologists, and gastroenterologists should be cognizant of this unusual presentation of hepatocellular carcinoma in a Native American. Such knowledge may help improve early diagnosis and survival.

## Background

Hepatocellular carcinoma (HCC) is a common malignancy in Southeast Asia. HCC and respiratory system cancers are the most common cause of cancer-related deaths in Taiwan [1, 2]. HCC is the fourth highest cause of cancer-related death in Japan [3] and the fifth most common cancer diagnosed in Korea [4]. Due to better diagnosis, improvements in management, and prolonged survival of patients, an increasing number of patients are diagnosed after extrahepatic spread. The most common sites of metastases are the lungs, lymph nodes, and bones [5]. While axial skeletal metastasis is common in HCC, initial presentation as spinal cord/root compression is extremely rare, and it may cause paralysis and bowel and bladder dysfunction. Early disease-focused treatment includes radiotherapy and surgery, which play a crucial role in decreasing spinal cord compression and improving quality of life. To the best of our knowledge, there are 33 cases published to date (Table 1). We did not find any reports of HCC presenting as spinal cord/root compression in Native Americans.

**Table 1:** Summary of cases of hepatoma presenting with spinal cord compression

Case #	Year of publication	Authors	Age (years)	Sex	Race	Underlying liver disease	Activity of disease	Presenting symptom
1	1989	Omura *et al.* [24]	57	M	Paper from Japan	Not reported	Not reported	Paraplegia
2–17	1992	Lee [12]	26–59	M/F	n/a (paper from Taiwan)	75% (12/16) hepatitis B positive	Not reported	- Pain/weakness in the distribution of thoracic/lumbar spine – 8 cases - Arm weakness – 2 cases - Scalp mass – 3 cases - Right hemicranias – 1 case - Diplopia – 1 case - Dysarthria – 1 case
18	1993	Kantharia *et al*. [25]	45	M	n/a (paper from, Syracuse, NY state)	Hepatitis C, hepatitis B, and alcoholic liver disease with cirrhosis	Not reported	Low back pain
19	1997	Yang *et al.* [26]	37	M	n/a (paper from Hong Kong)	Hepatitis B	Not reported	Low back pain
20	1997	Yang *et al.* [26]	47	F	n/a (paper from Hong Kong)	Not reported	Not reported	Low back pain
21	1998	Razana *et al*. [27]	77	M	Malay (Asian)	Alcoholic with liver cirrhosis	ALT and AST were elevated	Right lower limb weakness and paresthesia
22	1998	Razana *et al.* [27]	68	M	Malay (Asian)	Not reported	Not reported	Sudden onset of lower extremities paraparesis
23	2003	Po *et al*. [28]	60	M	n/a (paper from Taiwan)	Hepatitis B and C	Remission	Low back pain
24	2005	Garcia and Castillo [29]	49	M	Not reported	Alcohol abuse, hepatitis B and C negative	Not reported	Low back pain
25	2006	Doval *et al*. [30]	55	M	Not reported (paper from China)	Hepatitis B	Remission	Low back pain
26	2006	Doval *et al*. [30]	70	M	Not reported (paper from China)	Alcoholic, hepatitis B and C negative	Not reported	Chest pain
27	2006	Doval *et al*. [30]	62	M	Not reported (paper from China)	Nonalcoholic, hepatitis B and C negative	Not reported	Pain in the neck and low back
28	2011	Vargas *et al*. [31]	50	M	Not reported (paper from USA)	Alcohol abuse, hepatitis B	AST 146 U/L, ALT 84 U/l	Low back pain
29	2014	Nangolo *et al*. [5]	46	M	Namibian (Africa)	Alcoholic hepatitis and hepatitis B	AST 180 IU/l and ALT 70 IU/l	b/l leg weakness
30	2014	Vallianou *et al*. [32]	79	M	n/a (paper from Greece)	Hepatitis B (not on medications)	Not mentioned	Upper extremity muscle pain and paresthesia
31	2015	Hwang *et al*. [4]	61	M	n/a (paper from South Korea)	Hepatitis B	AST 418 U/l ALT 594 U/l HBV DNA 1890769 copies/mL	Upper extremity weakness and tingling
32	2016	Sangli *et al*. [14]	49	M	Emigrant from Ghana	Hepatitis B not on medications	ALT and AST WNL	Left lower extremity weakness and numbness
33	2017	Ayyadurai *et al*. [13]	58	M	n/a (paper from Bronx, USA)	Hepatitis C and alcohol abuse	LFTs WNL	Neck pain
34	2017	Our patient	64	M	Native American	Hepatitis C (treated, no viral load detected)	ALT 505, AST 210, bilirubin 1.5	Lower back pain and numbness
35	2017	Our patient	70	M	Native American	Hepatitis C Ab positive. RNA not detected	ALT 90, AST 104	Upper back pain and numbness of right foot

*Ab* antibody, *ALT* alanine aminotransferase, *AST* aspartate aminotransferase, *b/l* bilateral, *F* female, *HBV* hepatitis B virus, *LFTs* liver function tests, *M* male, *n/a* not available, *WNL* within normal limits

We report the cases of two Native American patients who presented with spinal cord compression secondary to HCC metastasis associated with hepatitis C infection. In one of these cases, the hepatitis C infection had been successfully controlled. Hepatitis C titers were undetectable for a year before the patient developed spinal cord compression. We did not find any literature reporting HCC presenting with spinal cord compression in Native Americans.

## Case presentation

### Case 1

**Fig. 1:**
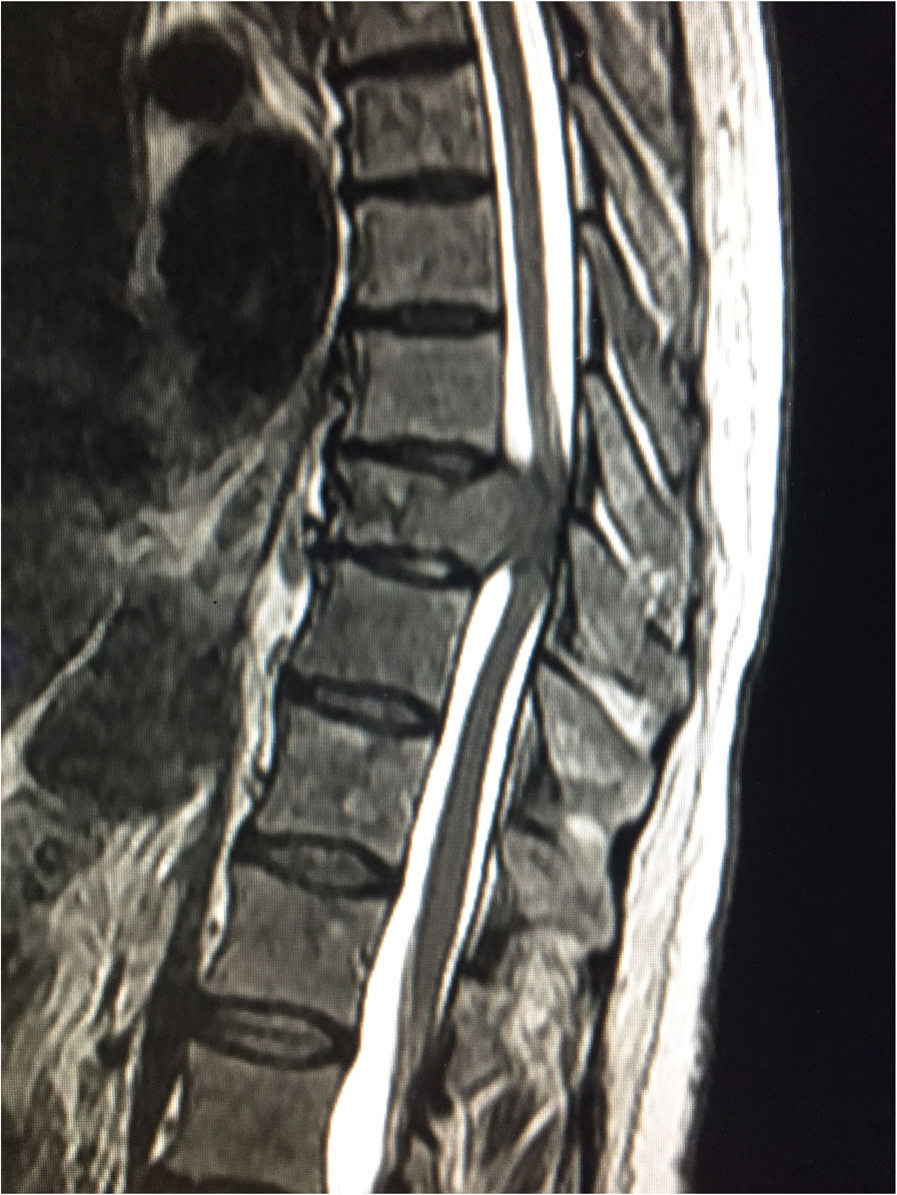
Magnetic resonance imaging of the thoracic spine, sagittal short T1 inversion recovery sequence. Demonstrates T11 metastasis with pathologic fracture and retropulsion of the vertebral body into the spinal canal with thoracic cord compression

**Fig. 2:**
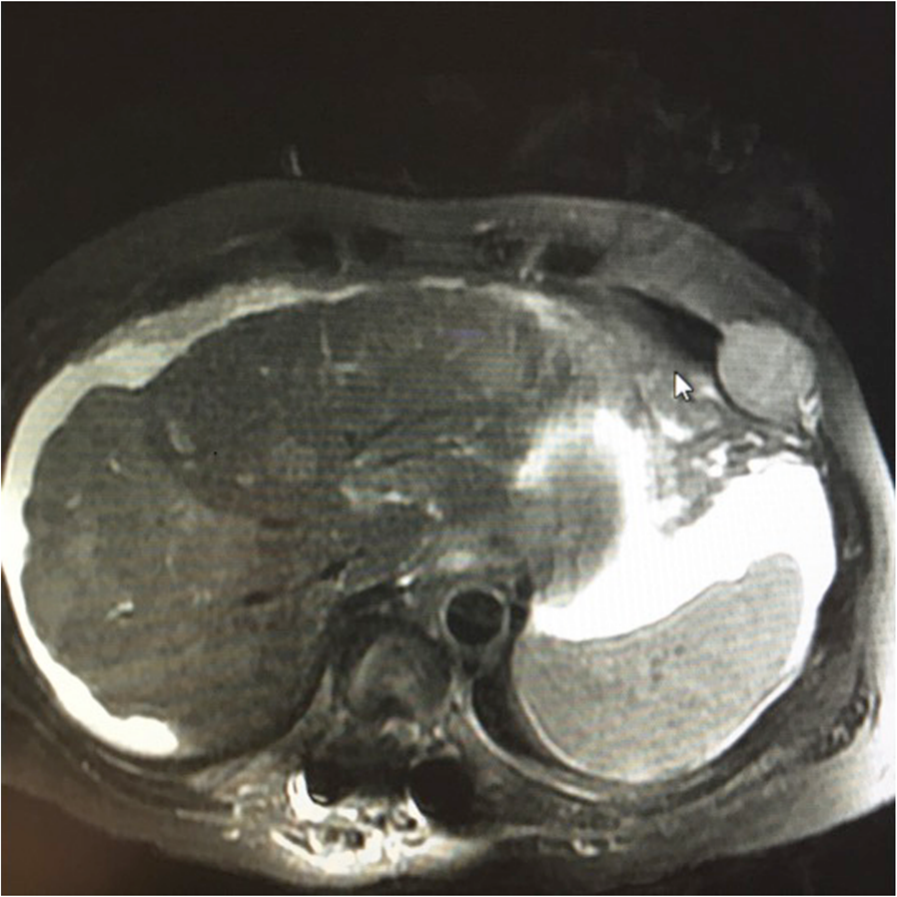
Magnetic resonance imaging of the thoracic spine. Axial T2 sequence demonstrates right chest wall and thoracic spine mass with tumor invasion into the spinal canal and thoracic cord compression at T6

**Fig. 3:**
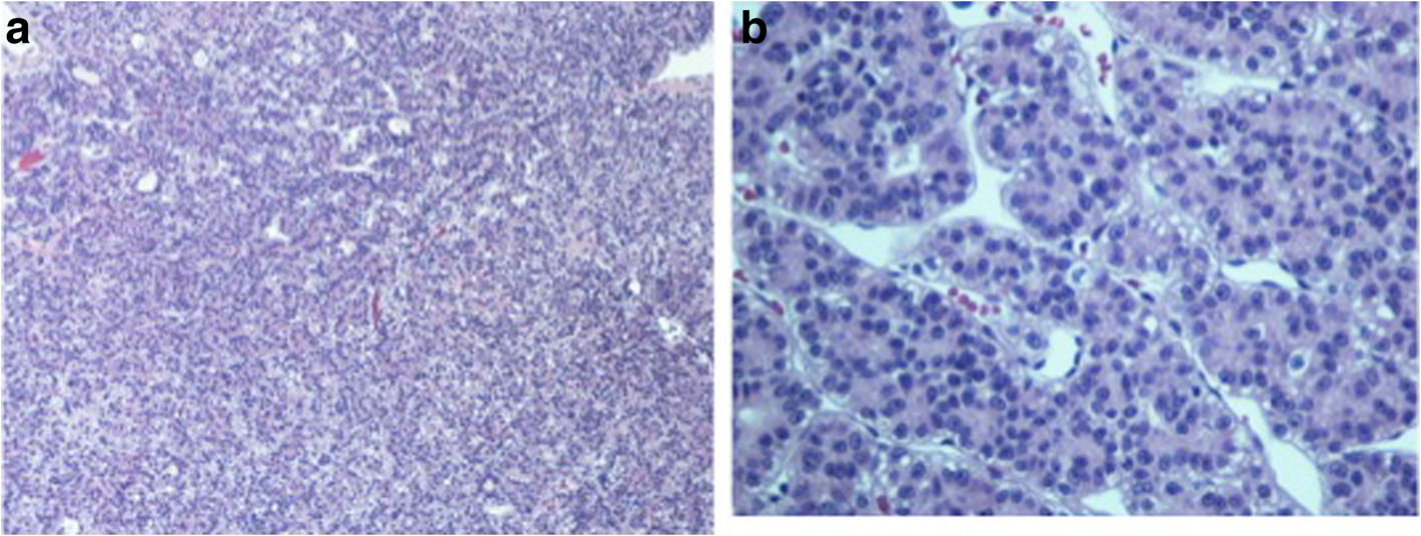
**a** Hematoxylin and eosin 10×; **b** high power view, section of the specimen shows solid or packed papillary pattern with fibrovascular cords

**Fig. 4:**
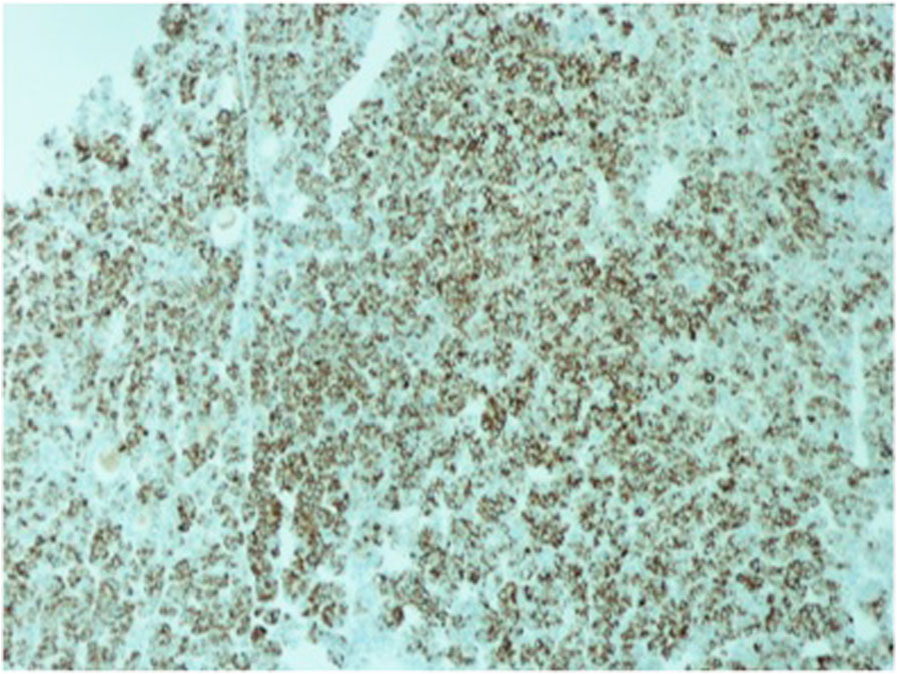
Immunostaining of Hep Par-1 and glypican-3. 10×: Hep Par-1 and glypican-3 both show tumor cells to be diffuse strongly positive stains

A 64-year-old Native American man presented with worsening lower back pain, and numbness and tingling radiating from his belly button down both legs. At the time of admission, he reported gradually increasing weakness in both legs for 3 days that led to an inability to walk. His past medical history is significant for hepatitis C for many years, which led to liver cirrhosis. His past surgical history is significant for a previously repaired umbilical hernia. His family history included breast cancer (sister) and lung cancer (mother). He smoked cigarettes for 1–2 years in the 1980s, but it is unknown how many cigarettes he smoked per day. In addition, he was a former heroin abuser. He never consumed alcohol. He worked as a manager in the laundry department in a hospital. Family members deny any exposure to asbestos. An ultrasound of his liver 1 year prior to the current presentation reported coarse echotexture, suggestive of underlying cirrhosis. Several years earlier, he had not responded to interferon and ribavirin treatment. However, 1 year before presentation, he did respond to ledipasvir/sofosbubir (Harvoni) treatment. Although he cut the treatment short to just 5 weeks, a recent hepatitis viral test detected no hepatitis C ribonucleic acid (RNA). He had hepatitis C virus (HCV) RNA genotype 1a. He was a prior intravenous drug user and was in a methadone program. Home medications were as follows: nadolol, spironolactone, bumetanide, and methadone. On admission, his blood pressure (BP) was 109/67 mm Hg, heart rate (HR) 57 beats per minute, and temperature 36.6 °C. A physical examination had the following results: no jugular venous distention, his lungs were clear to percussion and auscultation, his heart sounded normal, there were no murmurs, his abdomen was slightly distended, his spleen and liver were not palpable, and some spider angioma was noted on his skin. On neurological examination: he was alert and awake; he was oriented to time, his name, and his location; and his cranial nerves were grossly intact. While no gait disturbance was observed, marked weakness of his lower extremities and swelling over the T9 area of his spine were found. He had a blood urea nitrogen (BUN) of 66 mg/dL, creatinine of 2.8 mg/dL, alkaline phosphatase of 505 U/L, aspartate aminotransferase of 210 U/L, alanine aminotransferase of 66 U/L, and total bilirubin of 1.5 mg/dL. Magnetic resonance imaging (MRI) of his thoracic and lumbar spine revealed a pathologic fracture at T11 with retropulsion and severe cord compression (Fig. 1) and right chest wall and thoracic spine mass with tumor invasion into the spinal canal and thoracic cord compression at T6 (Fig. 2). In addition, numerous metastatic lesions in his thoracic and lumbar spine were noted. A MRI scan of his chest/abdomen and pelvis without contrast was performed and revealed a large right liver mass and multiple lesions in his ribs, spine, and mediastinum, suggestive of metastatic disease. He was started on intravenously administered steroids. Surgical spinal cord decompression and stabilization/fusion of his spine was performed. Pathology results of an intervertebral disc and the T9 vertebral body reported metastatic carcinoma favoring HCC (Fig. 3). Tumor cells were positive for Hep Par-1 and glypican-3 (Fig. 4), and negative for cytokeratin (CK) 7, CK20, thyroid transcription factor 1 (TTF-1), inhibin, OCT3/4, prostate-specific antigen (PSA), prostatic specific acid phosphatase (PSAP), renal carcinoma marker (RCC), and PAX8. Subsequently, he was treated with radiation to the T11 spine lesion and was scheduled to begin radioembolization with yttrium-90, but his condition deteriorated, and he died 2 months after diagnosis. An autopsy was not performed.

### Case 2

**Fig. 5:**
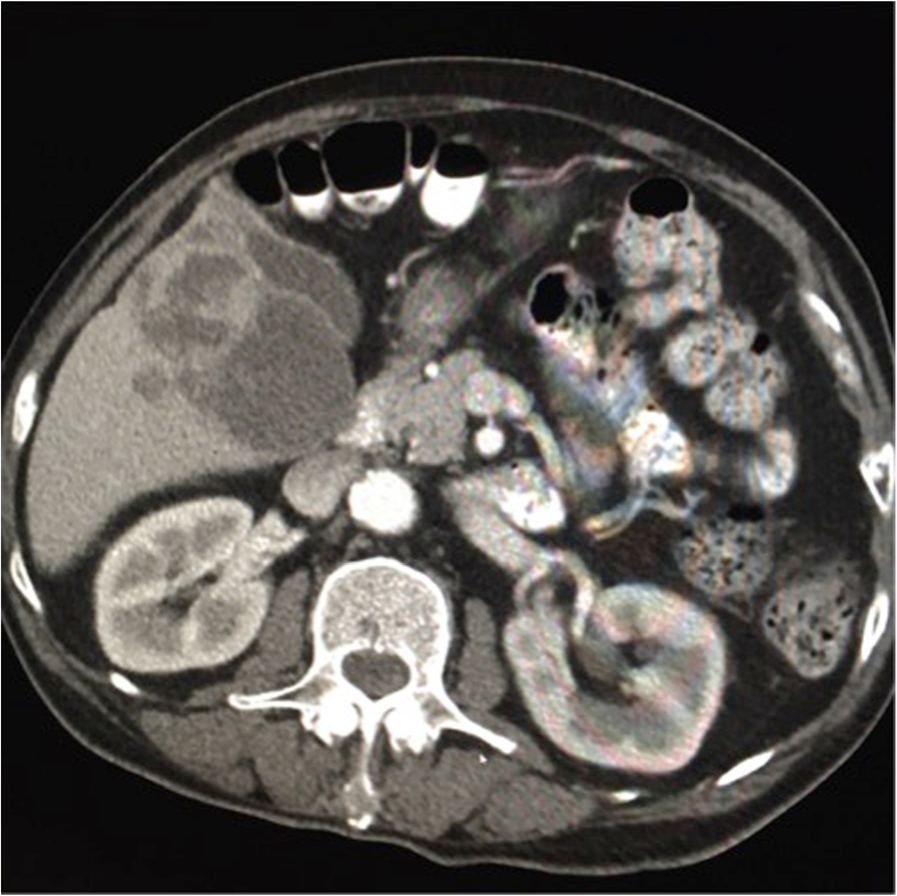
Computed tomography of the abdomen and pelvis with contrast demonstrates a right hepatic mass with a cystic component. The gallbladder is medial to the mass

The second case involved a 70-year-old Native American man presenting with upper back pain and numbness of his right foot for approximately 10 days. The symptoms had worsened, and he noticed some difficulty with walking. He did not have any past medical or surgical history. He was a former tobacco smoker and stopped smoking approximately 20 years ago, but it is unknown how many packs or cigarettes per day he smoked. He drank alcohol very rarely and not significantly. He did not have family history of any significance. He was never on medications until he was diagnosed as having HCC. He worked at a warehouse in the past. His job position was unknown. He has no known environmental or drug allergies. On admission, his BP was 166/119 mm Hg, HR was 97 beats per minute, and temperature was 36.7 °C. His physical examination had the following results: he was normocephalic, he had a non-traumatic skull, he had normal hearing, he had no nasal discharge, his chest wall movement was symmetric, his breath sounds were clear, he had no rales/wheezing, his HR was within the normal limit and had regular rhythm with no murmurs or thrills, his abdomen was soft with no distension, there was no palpable mass, there was no hepatomegaly or splenomegaly, a bilateral pedal pulse was present, there was no visible joint swelling, his skin was warm to the touch, he had normal color, and he had no rash/ulcers. A neurological examination had the following results: he was alert and awake; he was oriented to time, his name, and his location; his cranial nerves were grossly intact; he had no gait disturbance or motor deficits; his superficial reflexes were intact; a slight decrease in sensation over his right lower extremity was noted. Abnormal laboratory results were as follows: aspartate aminotransferase level of 104 U/L and alanine aminotransferase level of 90 U/L. CT of his chest and abdomen revealed a 10.0 × 8.3 × 7.4 cm soft tissue mass with associated osseous destruction involving the posterior right fourth, fifth, and sixth ribs and adjacent thoracic vertebral bodies, with significant soft tissue extension into the spinal canal and evidence of spinal cord compression. An additional lesion of the left iliac wing measuring 3.6 cm was noted. There were numerous enhancing lesions throughout his liver that were suspicious for primary versus metastatic disease (Fig. 5). No signs of cirrhosis were detected on CT images. Further laboratory tests showed alpha-fetoprotein (AFP) levels of 98,884.0 ng/mL. Carcinoembryonic antigen (CEA) and cancer antigen (CA) 19-9 levels were slightly elevated. Hepatitis C RNA genotype 1a was detected. Hepatitis C RNA was 6.73 log IU/mL by reverse transcriptase polymerase chain reaction (RT-PCR).

**Fig. 6:**
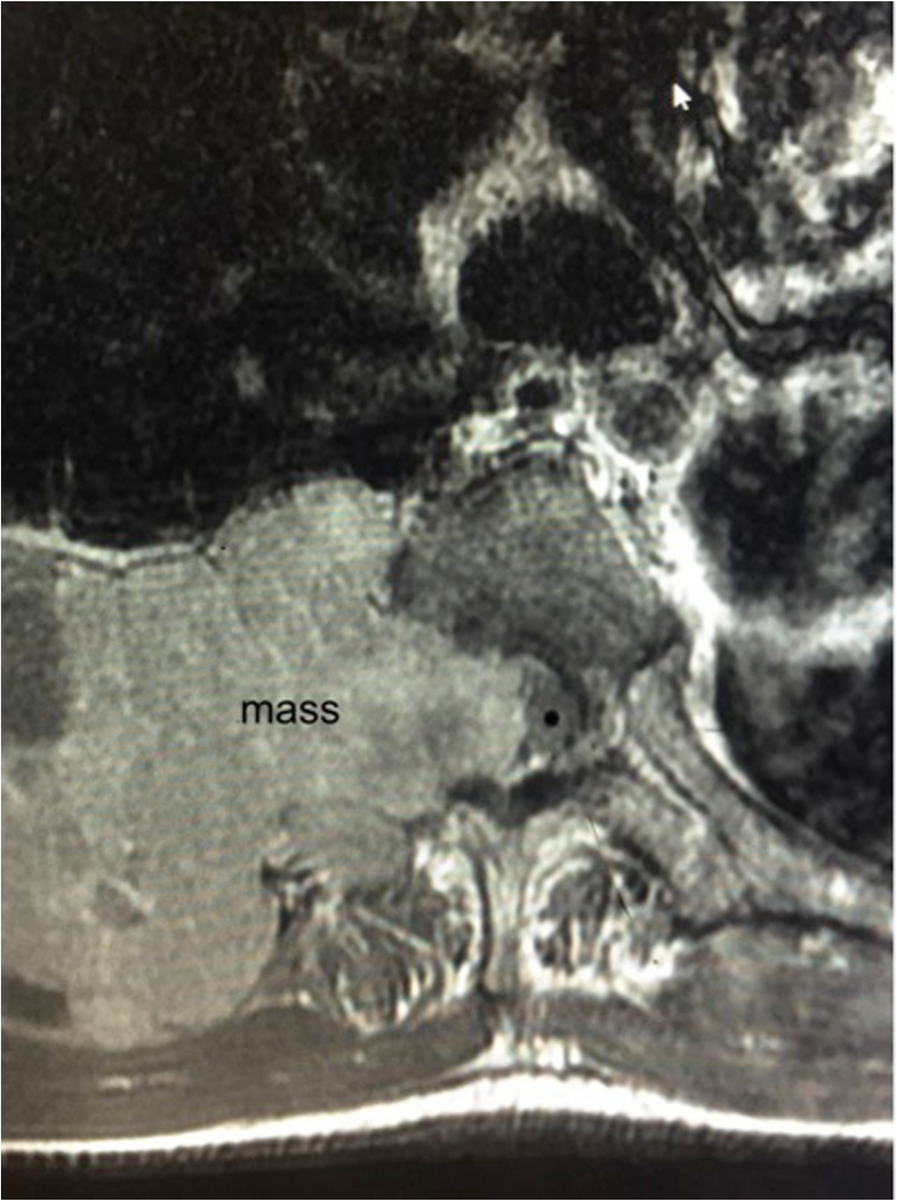
Magnetic resonance imaging of the thoracic spine, sagittal short T1 inversion recovery sequence. Demonstrates metastases to the mid-thoracic spine, T5, T6, and T7, with tumor invasion into the spinal canal and cord compression from T5 to T7

**Fig. 7:**
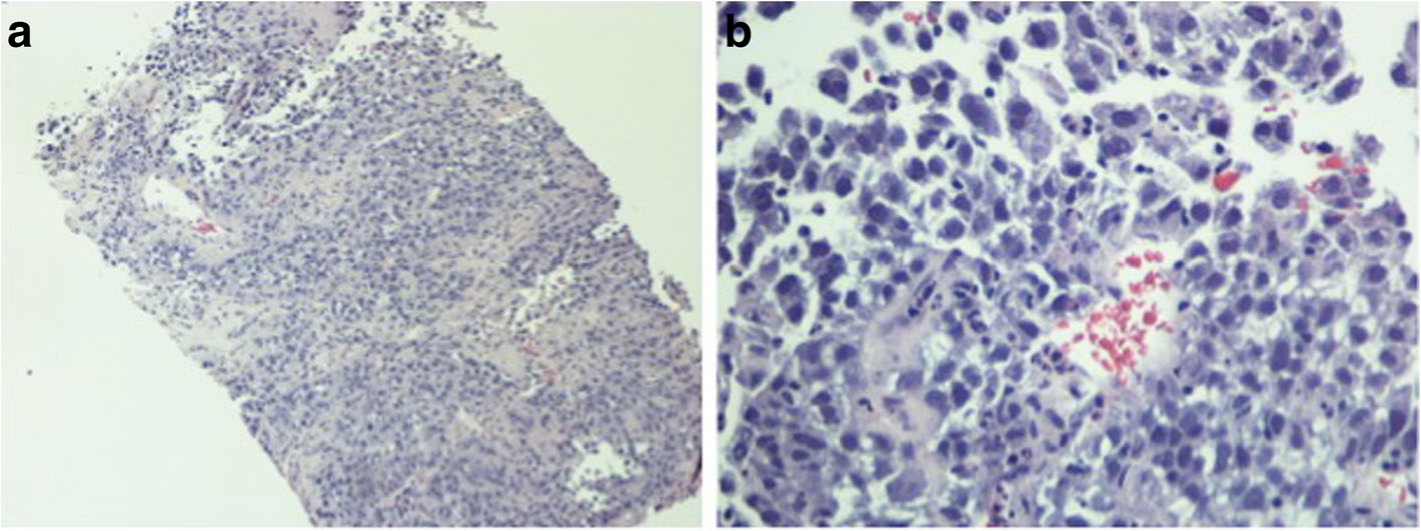
**a** Hematoxylin and eosin 10×. Section of the specimen demonstrates infiltrative tumor cells in fibrous tissue; **b** high power view. Tumor cells with marked cytological pleomorphisms. There is abundant pink or clear cytoplasm. Nuclei are round to oval with irregular nuclear contour and hyperchromatic chromatin. Mitoses are frequently identified. Based on morphologic features, diagnosis of poorly differentiated carcinoma is suspected

**Fig. 8:**
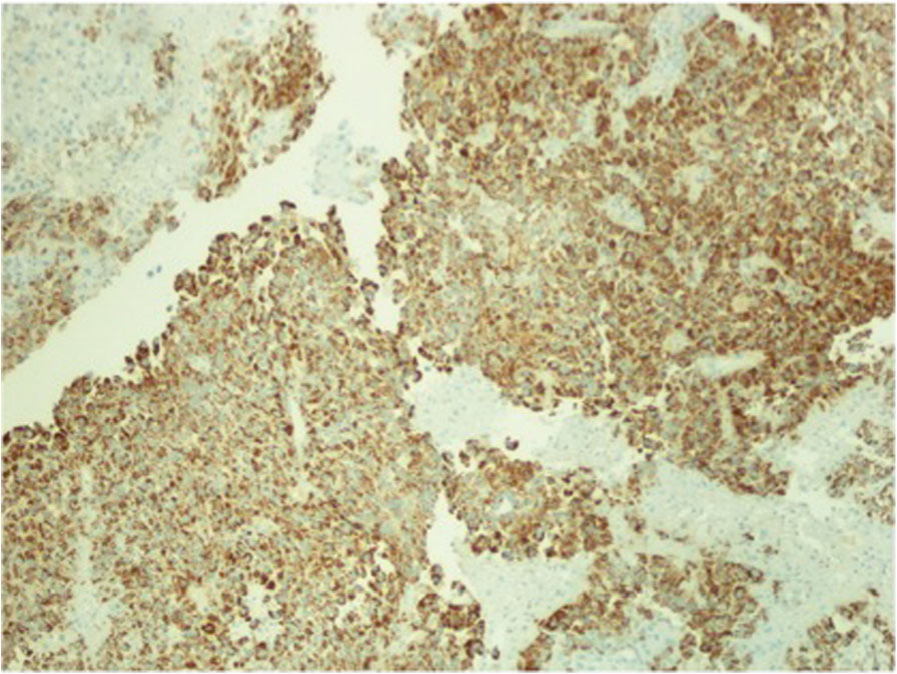
Case 2. Immunostaining of Hep Par-1 and glypican-3. 10×: Hep Par-1 and glypican-3 both show tumor cells to be strongly positive diffuse stains

An MRI of his thoracic spine was performed and revealed a large, posterior, left chest wall mass measuring 10.0 × 6.7 × 9.5 cm, with associated osseous destruction of the underlying right posterior fourth through seventh ribs and adjacent vertebral bodies, with complete obstruction of the right foramen and significant involvement of his spinal canal, causing spinal cord compression (Fig. 6). A biopsy of the soft tissue mass was performed and showed a metastatic, poorly differentiated carcinoma favoring hepatocellular origin (Fig. 7). Tumor cells were positive for hepatocyte, glypican-3, and pan-CK (Fig. 8), and focally positive for CK20 and CEA but were negative for CK7 and TTF-1, CA 19-9, p63, CDX2, and OCT3/4. Because of the poorly differentiated histology and atypical presentation, he was treated with oxaliplatin plus fluorouracil/leucovorin [6]. After radiation of T5–T7 and a subsequent decrease in AFP levels, he eventually refused further therapy and was placed on hospice care. He died 6 months after diagnosis. An autopsy was not performed.

## Discussion

Metastasis to the spinal cord is a very unusual and rare presentation of HCC. To the best of our knowledge, only 33 such cases have been published to date (Table 1). Of those 33 patients, 21 had evidence of previous hepatitis B infection, and two of them had hepatitis C as well. One patient was positive for hepatitis C and had a history of alcohol abuse. Three patients had a history of alcohol abuse without evidence of viral hepatitis infection. One patient who was not an alcoholic was negative for both hepatitis B and C. Data on hepatitis serologies were not available for seven of the cases. The incidence of bone metastasis varies from 1% to 20% [7]. Due to improved diagnosis and therapeutic modalities for HCC, more cases of extrahepatic metastases, especially bone metastases, have been detected in recent years. We report two cases of Native Americans with HCC presenting with a spinal cord compression. We could not find any previous publications of HCC presenting with spinal cord compression in Native Americans. One of our cases had undetectable hepatitis C RNA levels at the time of HCC diagnosis. In another case, there were no signs of liver cirrhosis when the patient was diagnosed as having HCC.

HCC is the most common primary hepatic cancer and is an uncommon cancer in Western countries, including the USA. The overall incidence in the USA is 0.21–0.57%, but the incidence is higher in Asia and sub-Saharan Africa. In Japan, the relative frequency of HCC during autopsies is 2.57–4.8% [7]. It ranks as the fourth highest cause of cancer-related death in Japan [3]. The high incidence of HCC is largely driven by the high burden of hepatitis B and HCV infection in these regions [3, 5], with hepatitis B infection being found in 75–80% of patients with HCC [8, 9]. In the Asian population, the rate of seropositivity for hepatitis B surface antigen (HBsAg) approaches 100% in children with HCC compared with 70–80% in adults with HCC [9].

An epidemiological study of HCC in Taiwanese children aged 6 to 14 began after the launch of a nationwide vaccination program in 1984. The average incidence of HCC declined from 0.70 per 100,000 children for the period of 1981 to 1986 to 0.36 per 100,000 children for the period of 1990 to 1994 [9]. In many developed nations, including the USA, HCV infection accounts for more than half of HCC cases, in contrast to Asian countries [10].

While the pathophysiology of HCC in hepatitis C or B infection is unclear, the chronic inflammatory process in the liver may play a significant role. The liver inflammatory processes stimulate growth, repair, and restoration of normal liver architecture. When liver inflammation becomes chronic, the balance of damage versus regeneration is impaired and stimulates the formation of excess fibrotic tissue. In the long term, liver inflammation leads to cirrhosis, which is characterized by abnormal liver architecture and function. Cirrhosis leads to end-stage liver disease, hepatic failure, and liver cancer [10]. HCC can also occur in non-cirrhotic patients, particularly in patients with hepatitis B infection [11].

The risk factors for HCC include Asian and African race, cirrhosis of the liver, and hepatitis B or C infection. HCC is one of the more aggressive neoplasms, with metastatic potential mainly targeting the lungs, lymph nodes, bone, and adrenal glands. Most patients with HCC present with hepatomegaly, right upper quadrant pain, and/or abdominal mass [12]. While bone metastasis is reported to occur in cases of HCC, its presentation as spinal cord compression is extremely unusual [5, 12–14].

In Taiwan, HCC is the fifth most frequently diagnosed cancer and second highest cause of cancer-related mortality [15]. In the USA and other Western countries, HCC accounts for less than 2% of all neoplasms and is often related to hepatitis C infection or alcohol intake. In Asia, where hepatitis B virus is endemic, HCC is commonly associated with hepatitis B infection [16]. In the USA, chronic HCV is the leading risk factor for HCC [17].

According to the population-based Surveillance Epidemiology and End Results registry data, the overall HCC incidence rate is approximately 6 per 100,000 in the USA. It is more common in Asian men [18]. However, the largest increase is occurring among Hispanics, followed by African Americans and non-Hispanic whites, with the lowest increase occurring among Asians [17, 18]. The incidence and mortality of HCC is highest in Southern USA (that is, Texas, Louisiana, and Mississippi) [18]. HCV infection is associated with a 15-fold to 20-fold increased risk of HCC compared with individuals who are HCV negative. Following establishment of HCV-related cirrhosis, HCC develops at an average annual rate of 1–8% [18]. The presence of any level of HCV viremia is a strong risk factor for HCC compared to the absence of viremia [18]. While a dose–response relationship between hepatitis C RNA level and liver cirrhosis has been reported [19], viral load is not associated with HCC [17]. Patients who are HCV positive with advanced fibrosis who clear viremia with antiviral treatment have a reduced, though not eliminated, risk of HCC [17]. HCC can occur even after more than 10 years have passed since successful HCV clearance [20]. It is suggested that HCV leads to irreversible changes in cellular signaling via mechanisms such as epigenetic activation or imprinting, which continue to drive carcinogenesis even after viral clearance [20]. Currently, the widespread implementation of novel direct-acting antivirals, which target the viral protease, polymerase, or nonstructural proteins, achieves 90% of the sustained virologic response. However, additional large studies with long-term follow-up are required to determine the HCC incidence rate after HCV eradication. Our first case demonstrates that even with eradication of hepatitis C infection, hepatoma still developed after many months.

The incidence of HCC in Native Americans was 3.5–6.6 per 100,000 [20], which is slightly higher than that in whites (2.6–3.5 per 100,000) and slightly lower than that in African Americans (4.2–7.0 per 100,000) and Hispanics (4.8–8.0 per 100,000) [21]. Stewart *et al.* showed that cause-specific survival in Native Americans was slightly higher (44.6 weeks, 95% confidence interval) than it was in whites (42.4 weeks, 95% confidence interval) and African Americans (36.3 weeks, 95% confidence interval) [22]. Xu *et al*. reported that in the USA, Asian patients demonstrated the highest overall survival of 15 months compared with white, black, and Native American patients who had an overall survival of 11 months, 9 months, and 12 months, respectively (all *p* < 0.05) [23].

## Conclusions

Most of the reported cases of HCC presenting with spinal cord compression have been reported in Asian countries, and most were associated with hepatitis B infection (Table 1). Our cases represent two patients of Native American origin, which, to the best of our knowledge, have never before been published. Both patients were hepatitis C positive and negative for hepatitis B infection. Physicians should be aware of the differentials of spinal cord lesions, including HCC, especially in patients suffering from hepatitis C, hepatitis B, or alcoholic liver disease. Patient survival could improve if HCC is diagnosed earlier.
